# Imaging Evaluation of Insertion Point Accuracy in Retrograde Intramedullary Femoral Nailing

**DOI:** 10.1155/2022/6068490

**Published:** 2022-10-28

**Authors:** Miao He, Shanshan Tian, Jian Liu, Xu Deng, Jiangxia Xiang

**Affiliations:** ^1^Department of Orthopedic Surgery, Chongqing Emergency Medical Center (Chongqing University Central Hospital), No. 1 Jiankang Road, Chongqing 400010 ., China; ^2^Department of Prehospital Emergency, Chongqing Emergency Medical Center (Chongqing University Central Hospital), No. 1 Jiankang Road, Chongqing 400010 ., China; ^3^Department of Traumatology, Chongqing Emergency Medical Center (Chongqing University Central Hospital), No. 1 Jiankang Road, Chongqing 400010, China

## Abstract

**Objective:**

When compared with visual retrograde intramedullary nail placement in the femur, fluoroscopic retrograde intramedullary nail placement in the femur improved the accuracy of insertion.

**Methods:**

Ninety-six patients treated with retrograde intramedullary nailing of the femur for femoral fracture were included in this retrospective case-control study, including 48 patients treated with nailing under direct vision and 48 patients treated with nailing under fluoroscopy. Influencing factors potentially associated with the deviation of the needle insertion point on the coronal and sagittal planes (including the needle insertion method, use of limited open reduction, side, intramedullary nail diameter, mechanism of injury, and fracture classification) were analyzed univariately; then, the variables with a *p* value < 0.20 on univariate analysis were included in the linear regression equation to assess the independent factors associated with needle insertion point deviation.

**Results:**

On the coronal plane, the insertion point deviation in the visual nail placement group (1.11 ± 4.08 mm) was not significantly different (*p* = 0.13) from that in the fluoroscopic nail placement group (−0.44 ± 3.48 mm); on the sagittal plane, the insertion point deviation in the visual nail placement group (4.91 ± 4.67 mm) was significantly greater than that in the fluoroscopic nail placement group (2.08 ± 2.97 mm) (*p* < 0.01). Visual nail placement was a risk factor for insertion point deviation on the sagittal plane compared with fluoroscopic nail placement (*β* = −0.84, *p* < 0.01).

**Conclusion:**

Compared with visual nail placement, fluoroscopic nail placement improves the accuracy of insertion on the sagittal plane, with no difference between the two methods on the coronal plane. These findings indicate that surgeons should exercise more caution when placing nails under direct vision.

## 1. Introduction

With as many as 2.9 million femoral fractures per year, the global burden is high [1]. The recommended treatment for most femoral fractures is intramedullary nailing [2, 3], which can be mainly divided into prograde and retrograde techniques. Retrograde intramedullary nailing via the intercondylar notch was developed to provide solutions to difficulties in the treatment of ipsilateral pelvic or acetabular fractures, polytrauma, and distal and diaphyseal femoral fractures [4–6]. Retrograde intramedullary nailing has been found to yield a high rate of fracture healing, a short healing time, and a low rate of deformity after healing [7, 8].

Currently, confirmation of the entry point during retrograde intramedullary femoral nailing is mainly performed by intraoperative fluoroscopy. On imaging, the ideal entry point is located medially (coronal plane) in the intercondylar fossa of the femur, just above Blumensaat's line (sagittal plane) [9]. However, when a medical institution lacks an intraoperative C-arm machine or the surgeon is confident in his or her own technique, he or she may perform insertion under direct vision with no fluoroscopic assistance. When confirmed under direct vision, the insertion point is located approximately 1 cm anterior to the posterior cruciate ligament in the center of the intercondylar groove [10].

Selection of the retrograde femoral nail insertion point is important. Incorrect intramedullary nail placement, i.e., in which the nail does not coincide with the anatomical axis with the femur, not only can lead to poor fracture alignment and cortical bone damage but can also endanger the soft tissue structures and articular cartilage within the knee joint [10].

The main objective of this article was to determine the accuracy of insertion by reviewing images of the insertion point achieved under direct visual nail placement and fluoroscopic nail placement and comparing them with the ideal retrograde intramedullary femoral nail insertion point on imaging, as there is no relevant literature describing a comparative analysis of the insertion point under direct visual and fluoroscopic nail placement. Our main null hypothesis was that there is no difference between the different methods.

## 2. Materials and Methods

### 2.1. Study Design

We performed a retrospective analysis of all patients with femoral fractures treated with retrograde intramedullary nailing from January 2016 to March 2022. We initially identified 124 patients; 15 patients with inadequate postoperative images and 13 patients in whom the tail of the retrograde intramedullary nail obscured the intercondylar fossa or Blumensaat's line of the femur were excluded, resulting in a total of 96 patients (55 OTA/AO type 33 and 41 OTA/AO type 32) [11], 48 patients treated with nail placement under direct vision and 48 patients treated with nail placement under fluoroscopy. One senior orthopedic surgeon performed the surgeries with direct visualization. Another senior orthopedic surgeon performed the procedures with fluoroscopic nailing.

Seven open fractures were treated with irrigation and debridement prior to final fixation. Six fractures included nondisplaced or slightly displaced intra-articular fractures that were fixed with compression screws prior to retrograde intramedullary nailing. All fractures were treated with the retrograde intramedullary nail (Smith & Nephew. Tennessee, USA) with a 3 to 4 cm long incision, usually just medial or central to the patellar tendon [12].

### 2.2. Radiography Measurements

**Figure 1:**
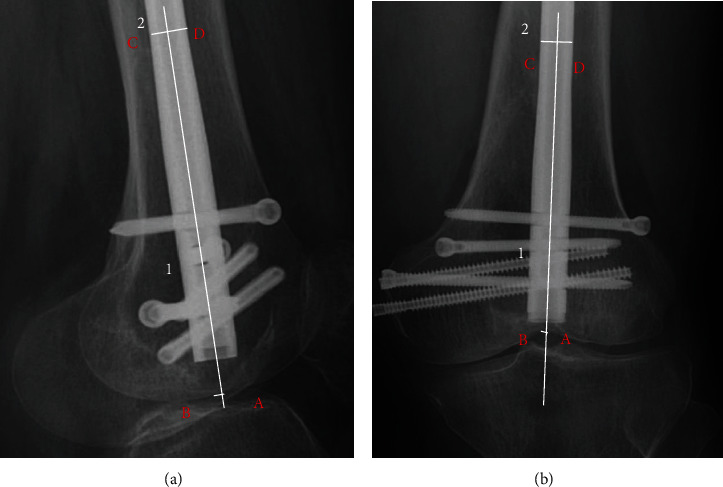
Measurement of deviation: (a) on the sagittal plane, line segment 1 is drawn across the axis of the intramedullary nail, and line segment 2 is drawn perpendicular to the axis of the intramedullary nail. Line segment 2 intersects the edges of the intramedullary nail at points C and D, respectively. The actual diameter of the intramedullary nail/measured length of CD = imaging ratio. The intersection of line segment 1 and the femoral condylar cortex is point A, and the ideal insertion point is point B. The deviation value = measured length of AB^∗^ imaging ratio. Point A is in front of Blumensaat's line; thus, the deviation value is positive. (b) Similarly, on the coronal plane, the deviation value = measured length of AB^∗^ imaging ratio. Point A is lateral to the middle of the intercondylar sulcus; thus, the deviation value is negative.

We performed a review of coronal and sagittal femoral X-rays prior to patient extraction to assess the accuracy of nail insertion. The ideal entry point is located medially in the intercondylar fossa of the femur on the coronal plane, just above Blumensaat's line on the sagittal plane. Measurements were calibrated using the diameter of the main intramedullary nail. The distance was measured (to the nearest 0.1 mm) between the ideal insertion point and the observed insertion point as described above. On coronal imaging, the measured value was positive if the entry point was medial to the intercondylar fossa and negative if the insertion point was lateral to the intercondylar fossa (Figure 1). On sagittal imaging, the measured value was positive if the insertion point was anterior to Blumensaat's line and negative if the insertion point was posterior to Blumensaat's line (Figure 1). All measurements were performed by 2 blinded reviewers (Liu J and Deng X) to determine the interobserver reliability for the entire cohort. To assess the intraobserver reliability, 1 reader (Liu J) repeated all measurements after 1 month.

### 2.3. Statistical Analysis

IBM SPSS 22.0 (IBM Corp. New York, USA) was used for statistical analyses. The significance level was set at 0.05. Continuous variables are expressed as *x* ± *s*. Mean and standard deviation estimation yielded a group size of 48 patients (alpha, 0.05; power, 0.8). The chi-square test was used to compare qualitative variables. The Kolmogorov–Smirnov test was used to test whether the variables followed a normal distribution. Influential factors potentially associated with the deviation of the needle insertion point (coronal plane) (including the needle insertion method, use of limited open reduction, side, intramedullary nail diameter, mechanism of injury, and fracture classification) were analyzed univariately using two independent sample *t*-tests; similarly, the factors associated with needle insertion point (sagittal plane) deviation were analyzed univariately using the Mann–Whitney U test. The variables with a *p* value < 0.20 on univariate analysis were then included in the linear regression equation to assess the independent factors associated with the needle insertion point deviation.

## 3. Results

Ninety-six patients were included in the final analysis. There were 48 patients with a mean age of 49.9 ± 18.6 years in the direct visual nailing group and 48 patients with a mean age of 51.1 ± 20.1 years in the fluoroscopic nailing group (*p* = 0.276); there were 29 men in the direct visual nailing group and 34 men in the fluoroscopic nailing group (*p* < 0.01). The intraobserver reliability for the deviation on the coronal plane was 0.80 (95% CI, 0.67-0.88), and the interobserver reliability for the deviation value on the coronal plane was 0.86 (95% CI, 0.76-0.91). The intraobserver reliability for the deviation on the sagittal plane was 0.86 (95% CI, 0.57-0.94), and the interobserver reliability for the deviation on the sagittal plane was 0.89 (95% CI, 0.83-0.93) (Table 1).

**Figure 2:**
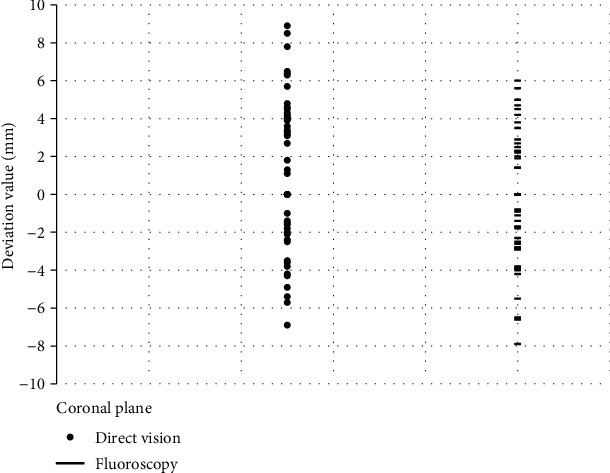
Scatter plot of nail insertion point deviation on the coronal plane.

In the coronal plane, there was no statistically significant difference (*p* = 0.13) in the deviation of the needle insertion point between the direct visual nail placement group (1.11 ± 4.08 mm) and the fluoroscopic nail placement group (−0.44 ± 3.48 mm) (Figure 2), and the use of limited open reduction, side, intramedullary nail diameter, mechanism of injury, and fracture classification were not found to be associated with needle insertion point deviation. The influencing factor (the needle insertion method) with a *p* value < 0.20 on univariate analysis was included in the linear regression equation. Compared with fluoroscopic nail placement, direct vision nail placement was not a risk factor for deviation of the nail insertion point on the coronal plane (*β* = −0.22, *p* = 0.05) (Table 2).

**Figure 3:**
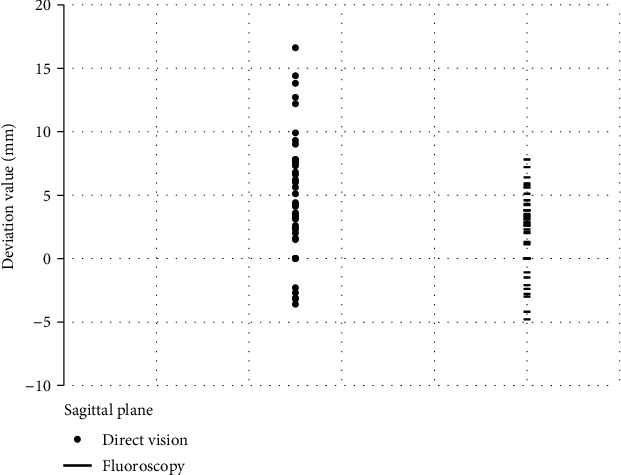
Scatter plot of nail insertion point deviation on the sagittal plane.

In the sagittal plane, there was no statistically significant difference (*p* < 0.01) in the deviation of the needle insertion point between the direct visual nail placement group (4.91 ± 4.67 mm) and the fluoroscopic nail placement group (2.08 ± 2.97 mm) (Figure 3), and the use of limited open reduction, side, intramedullary nail diameter, mechanism of injury, and fracture classification were not found to be associated with needle insertion point deviation. The influencing factors (the needle insertion method, side, and mechanism of injury) with a *p* value < 0.20 on univariate analysis were included in the linear regression equation. It was found that, compared with fluoroscopic nail placement, direct vision nail placement was a risk factor for deviation of the nail insertion point on the sagittal plane (*β* = −0.84, *p* < 0.01) (Table 3).

## 4. Discussion

When medical institutions lack an intraoperative C-arm machine or the surgeon is confident in his or her own technique, the surgeon may insert the needle under direct vision without the assistance of fluoroscopy. This procedure is similar to the placement of an intramedullary guide rod during total knee arthroplasty, which also does not require fluoroscopic assistance. Needle placement under direct vision is simple and does not require as much time as that under fluoroscopy, saving operation time. However, there is a lack of research on how accurately intramedullary nails are placed on the anatomical axis of the femur by this method.

Our results show that direct visual nail placement is a risk factor for insertion point deviation on the sagittal plane compared to fluoroscopic nail placement (*β* = −0.84, *p* < 0.01). We also found that the mean nail insertion point was further from the ideal point on the sagittal plane, and a larger standard deviation was observed for direct visual nail placement than for fluoroscopic nail placement. This finding further supports the higher sagittal deviation of fluoroscopic nail placement and serves as a reminder for surgeons to exercise more cautiously on the sagittal plane when placing nails under direct vision.

Interestingly, unlike on the sagittal plane, there was no statistically significant difference in the insertion point deviation between direct visual and fluoroscopic nail placement on the coronal plane (*p* = 0.13). Studies show that under direct visual placement, the anatomical location of the needle insertion point on the coronal plane is near the center of the intercondylar groove [8, 13, 14]. We hypothesize that with the limitations of the medial and lateral condyles, as well as easily recognizable anatomical features, the surgeon can accurately place the nail, resulting in no difference in the deviation between the direct and fluoroscopic nail entry points. However, the anatomical location on the sagittal plane is approximately 6-20 mm anterior to the posterior cruciate ligament [10, 13, 14]. This position shows large variability among individuals, which creates uncertainty in direct visual nail insertion and ultimately reduces the accuracy of nail insertion on the sagittal plane.

Accurate nail insertion has two main benefits. (1) It maintains or improves the adjusted position of the fractured ends: after the fractured ends are repositioned by various methods, proper intramedullary nail placement is needed to maintain or further improve the positioning. Precise intramedullary nail insertion allows the axis of the nail to match that of the femoral medullary cavity so that the nail does not crowd out or displace the fractured ends and can even help reset the proximal end of a fracture that does not coincide with the center of the distal medullary cavity. (2) Accurate nail insertion also avoids damage to intra-articular structures: an insertion point that is too anterior can lead to cartilage damage in the patellofemoral joint and impingement with the patella during flexion of the knee, while a point that is too posterior can jeopardize the beginning of the posterior cruciate ligament. A needle insertion point that is too medial or too lateral creates difficulty in completely seating the nail within the distal femur, leading to cartilage damage in the patellofemoral joint [10].

However, there are also some limitations to this study. High-quality radiographs were considered a prerequisite for inclusion in our study. However, in the process of imaging, rotation will inevitably occur, which will affect the accuracy of measurement. Walker et al. found that the width of the tibial plateau changes by 3% every 5 degrees of radiographic rotation [15]. Similarly, when the radiation angle changes, the measurement data of the femoral condyle will change accordingly. Moreover, the accuracy of our measurement has reached the millimeter level, which will amplify the inaccuracy of this measurement. In future research, we hope to find more accurate and reliable imaging technology to improve the accuracy of measurement. Additionally, not all the procedures were performed by the same surgeon, which contributes to statistical error. Finally, the sample size of the retrospective study was relatively small, which may have increased the probability of bias in the statistical results. Therefore, future prospective studies should include larger samples to validate our findings.

## 5. Conclusions

Compared to direct visual nail placement, fluoroscopic nail placement improves the accuracy of the entry point on the sagittal plane, with no difference between the two methods on the coronal plane. These findings indicate that the surgeon should exercise more caution when placing nails under direct vision.

## Figures and Tables

**Table 1:** Inter- and intraobserver reliability of all measurements^a^.

	Intraobserver reliability	Interobserver reliability
Deviation (coronal plane)	0.80 (0.67-0.88)	0.86 (0.76-0.91)
Deviation (sagittal plane)	0.86 (0.57-0.94)	0.89 (0.83-0.93)

^a^Values are presented as the intraclass correlation coefficient (95% CI).

**Table 2:** Univariate and linear regression analyses of all factors on the coronal plane.

	Coronal plane		Univariate analysis	Linear regression analysis			
	*n*	Deviation (mm)	*p* value^a^	Unstandardized *β*	Unstandardized SE	Standardized *β*	*p* value
*Method of needle insertion*							
Fluoroscopy	48	−0.44 ± 3.48	0.13	-1.55	0.77	-0.22	0.05
Direct vision	48	1.11 ± 4.08					
*Limited open reduction*							
Yes	42	0.16 ± 3.82	0.92				
No	54	0.48 ± 3.91					
*Side*							
Left	58	0.57 ± 3.64	0.41				
Right	38	−0.02 ± 4.19					
*Intramedullary nail diameter*							
≥11	49	0.69 ± 3.97	0.36				
<11	47	−0.04 ± 3.73					
*Mechanism of injury*							
High energy	61	−0.23 ± 3.82	0.92				
Low energy	35	1.32 ± 3.76					
*Fracture classification*							
A33	55	0.12 ± 3.77	0.55				
A32	41	0.63 ± 3.99					

^a^ *p* values are based on two independent-sample *t*-tests.

**Table 3:** Univariate and linear regression analyses of all factors on the sagittal plane.

	Sagittal plane		Univariate analysis	Linear regression analysis			
	*n*	Deviation (mm)	*p* value^a^	Unstandardized *β*	Unstandardized SE	Standardized *β*	*p* value
*Method of needle insertion*							
Fluoroscopy	48	2.08 ± 2.97	<0.01	-3.05	0.78	-0.84	<0.01
Direct vision	48	4.91 ± 4.67					
*Limited open reduction*							
Yes	42	4.06 ± 4.35	0.21				
No	54	3.05 ± 3.96					
*Side*							
Left	58	2.92 ± 3.78	0.14	-1.43	0.80	-0.17	0.08
Right	38	4.35 ± 4.57					
*Intramedullary nail diameter*							
≥11	49	3.06 ± 4.07	0.46				
<11	47	3.94 ± 4.22					
*Mechanism of injury*							
High energy	61	3.96 ± 4.27	0.13	1.52	0.82	0.18	0.07
Low energy	35	2.67 ± 3.84					
*Fracture classification*							
A33	55	3.33 ± 4.12	0.89				
A32	41	3.72 ± 4.22					

^a^ *p* values based on the Mann–Whitney *U* test.

## Data Availability

The datasets generated and/or analyzed during the current study are not publicly available due to their containing information that could compromise the privacy of research participants but are available from the corresponding author on reasonable request.
